# Analysis of Human Performance Deficiencies Associated With Surgical Adverse Events

**DOI:** 10.1001/jamanetworkopen.2019.8067

**Published:** 2019-07-31

**Authors:** James W. Suliburk, Quentin M. Buck, Chris J. Pirko, Nader N. Massarweh, Neal R. Barshes, Hardeep Singh, Todd K. Rosengart

**Affiliations:** 1 Michael E. DeBakey Department of Surgery, Baylor College of Medicine, Houston, Texas; 2 Department of Medicine, Baylor College of Medicine, Houston, Texas; 3 Center for Innovations in Quality, Effectiveness and Safety, Michael E. DeBakey Veterans Affairs Medical Center, Houston, Texas

## Abstract

**Importance:**

Potentially preventable adverse events remain a formidable cause of patient harm and health care expenditure despite advances in systems-based risk-reduction strategies.

**Objective:**

To analyze and describe the incidence of human performance deficiencies (HPDs) during the provision of surgical care to identify opportunities to enhance patient safety.

**Design, Setting, and Participants:**

This quality improvement study used a new taxonomy to inform the development and implementation of an HPD classifier tool to categorize HPDs into errors associated with cognitive, technical, and team dynamic functions. The HPD classifier tool was then used to concurrently analyze surgical adverse events in 3 adult hospital affiliates—a level I municipal trauma center, a quaternary care university hospital, and a US Veterans Administration hospital—from January 2, 2018, to June 30, 2018. Surgical trainees presented data describing all adverse events associated with surgical services at weekly hospital-based morbidity and mortality conferences. Adverse events and HPDs were classified in discussion with attending faculty and residents. Data were analyzed from July 9, 2018, to December 23, 2018.

**Main Outcomes and Measures:**

The incidence and primary and secondary causes of HPDs were classified using an HPD classifier tool.

**Results:**

A total of 188 adverse events were recorded, including 182 adverse events (96.8%) among 5365 patients who underwent surgical operations and 6 adverse events (3.2%) among patients undergoing nonoperative treatment. Among these 188 adverse events, 106 (56.4%) were associated with HPDs. Among these 106 HPD adverse events, a total of 192 HPDs (mean [SD], 1.8 [0.9] HPDs per HPD event) were identified. Human performance deficiencies were categorized as execution (98 HPDs [51.0%]), planning or problem solving (55 HPDs [28.6%]), communication (24 HPDs [12.5%]), teamwork (9 HPDs [4.7%]), and rules violation (6 HPDs [3.1%]). Human performance deficiencies most commonly presented as cognitive errors in execution of care or in case planning or problem solving (99 of 192 HPDs [51.6%]). In contrast, technical execution errors without other associated HPDs were observed in 20 of 192 HPDs (10.4%).

**Conclusions and Relevance:**

Human performance deficiencies were identified in more than half of adverse events, most commonly associated with cognitive error in the execution of care. These data provide a framework and impetus for new quality improvement initiatives incorporating cognitive training to mitigate human error in surgery.

## Key Points

**Question:**

What are the incidence and causes of human error as a source of adverse events associated with surgical care?

**Findings:**

In a quality improvement study including 5365 operations, 188 adverse events were recorded. Of these, 106 adverse events (56.4%) were due to human error, of which cognitive error accounted for 99 of 192 human performance deficiencies (51.6%).

**Meaning:**

Current systems-based approaches to improve surgical safety should be supplemented with additional focus on cognitive errors associated with surgical care.

## Teaser

This quality improvement study uses a novel taxonomy to analyze the associations of human performance deficiencies with surgical adverse events.

## Introduction

Adverse events in surgical care remain a frequent cause of injury or death and source of potentially avoidable health care expenditure in the United States.^1,2,3^ Preventable patient harm has persisted despite the reduction in complications that has been achieved during the past several decades through implementation of systems-based and team-based care approaches to ensuring patient safety.^4,5,6^ For instance, the deployment of crew resource management techniques adopted from the aviation and aerospace industries (eg, safety checklists) represents an important example of the application of a systems-based solutions to mitigate risk in health care delivery.^7,8,9^

The role of human error in causing adverse events in the delivery of medical care was highlighted in the 1990s by Reason,^10,11^ who broadly classified human error into failures of execution, termed *slips* and *lapses*, and failures of intention, termed *mistakes*. Nearly 3 decades after the seminal work of Reason,^10,11^ Leape,^9^ and others studying the effects of human error on health care delivery, to our knowledge, relatively little work has been undertaken to systematically analyze what we have termed *human performance deficiencies* (HPDs) and cognitive decision-making as a source of adverse events in medical and surgical care.^12,13,14,15,16,17,18^ Furthermore, most human error studies predate the current era of systems-based health care risk-reduction strategies.^12,13,14,15,16,17,18^ Analyses of the cognitive behavior of health care delivery teams and individual members of these teams in the modern era of systems-based safety nets for health care delivery may identify additional opportunities to enhance the safety of health care.

In this context, we conducted a prospective multisite study to define and characterize the role of human behavior in individual and team performance as a source of surgical complications. We hypothesized that HPDs are an important contributor to the occurrence of surgical adverse events. Importantly, compared with prior studies, this investigation included concurrent engagement of practitioners with first-hand knowledge of or involvement in index events and prospective application of an HPD classification tool that allowed detailed subclassifications of cognitive error and other HPDs as the root cause of surgical adverse events. These findings could provide a basis for new approaches to cognitive training for surgeons and other health care practitioners to enhance the safety of surgical care delivery, approaches similar to those used in other high-risk industries, such as the aerospace industry.^19^

## Methods

### Study Design

 Our study was reviewed by the Baylor College of Medicine Institutional Review Board and received a waiver of informed consent because it involved only the categorization of error in association with surgical adverse events without identifiable characteristics of patients, thus no personal health information was being collected as part of the study. The institutional review board further determined that the discussion of complications by surgeons was part of routine practice, thus also supporting exempt status. Our deidentified patient database included all surgical operations that were performed at our 3 affiliate adult hospitals (ie, a level I municipal trauma center, a quaternary care university hospital, and a US Veterans Administration hospital) and presented at weekly departmental morbidity and mortality (MM) conferences held at these institutions. Categorization and accrual of incidence of human error associated with adverse events began January 2, 2018, and concluded June 30, 2018. Data analyses were conducted from July 9, 2018, to December 23, 2018.

Our MM conferences include a comprehensive summary report of all surgical operations performed during the preceding week by the general surgery, acute care surgery, surgical oncology, cardiothoracic surgery, vascular surgery, and abdominal transplantation services. At these conferences, service-specific senior trainees make in-depth presentations of adverse events (Table 1) involving mortalities; major adverse events, as defined by the American College of Surgeons National Surgical Quality Improvement Program 30-day outcome complication definition^2,4^; or other nonroutine events.^10^ Case reviews include PowerPoint (Microsoft Corp) presentations of case data and patient course timelines obtained from the electronic medical record. Additional background information and data from the literature are included in these presentations as appropriate. The conferences are attended by all department faculty and residents on service at each hospital.

**Table 1.** Incidence of Adverse Events

Adverse Event	No. (%) (N = 188)
Mortality	14 (7.4)
Other major adverse events	
Wound event^a^	46 (24.5)
Iatrogenic surgical injury	23 (12.2)
Unexpected bleeding or transfusion	15 (8.0)
Venous thromboembolism^b^	12 (6.4)
Respiratory event^c^	11 (5.9)
Sepsis or septic shock	8 (4.3)
Urinary event^d^	7 (3.7)
Cardiac event^e^	6 (3.2)
Unplanned return to operating room	4 (2.1)
Neurologic event^f^	1 (0.5)
Other	41 (21.8)

^a^ Includes surgical site infection and wound dehiscence.

^b^ Includes deep venous thrombosis and pulmonary embolism.

^c^ Includes unplanned intubation, pneumonia, and the patient needing a ventilator more than 48 hours after the operation has ended.

^d^ Includes acute kidney injury and urinary tract infection.

^e^ Includes cardiac arrest and myocardial infarction.

^f^ Includes unintentional cerebrovascular injury, coma, or nerve injury.

### HPD Classification Tool and HPD Reporting System

To assist in identification and classification of human error in surgical complications, we developed an HPD classifier tool modeled on the work of Reason.^10,11^ This HPD classifier tool was developed using a systematic literature review that incorporated Preferred Reporting Items for Systematic Reviews and Meta-analyses (PRISMA) reporting guideline (eFigure 1 in the Supplement) to facilitate comprehensive and orderly synthesis of human error types, contexts, and themes. We then used this literature review as a basis to classify HPDs into 5 major categories of function related to cognitive, technical, and team dynamic behaviors (ie, planning or problem solving, execution, rules violation, communication, and teamwork).

Implementation of the HPD classifier tool in our study and formal entry into our database began after all surgeons were trained with the tool in human error taxonomy and categorization during an initial 2-week run-in period. The training began with a didactic tutorial performed by 1 of us (J.W.S.), including a PowerPoint-based visual education and review of examples of each of the 5 HPD classes (eFigure 2 in the Supplement). The run-in period incorporated discussion among 3 MM conference attendees (J.W.S., T.K.R., and a nonauthor) who formed an investigator study panel to obtain consensus adjudication and classification of HPD categories in test cases across study sites. This training was deployed to allow for the study to build real-time assignment and classification of HPD events at the weekly MM conferences.

### HPD Determination Process

Human performance deficiencies were identified and classified by service trainees in consultation with attending faculty participating in the specified operation, based on reporting classifications found in our HPD classifier tool. Immediately after their MM case presentations, the service trainees used an HPD classifier tool form to present these assignments of the preliminary HPD classifications for adverse events that occurred during the preceding week (eFigure 3 in the Supplement). After the presentation, a discussion was held among MM conference attendees and the investigator study panel to reach a consensus designation of 1 primary, causal adverse event per case and the classification of 1 or more HPDs (or a designation of no HPD) associated with that adverse event.

### Multiple HPD Proximate Cause Analysis

Morbidity and mortality case discussions were included in a situation, background, assessment, and recommendation narrative summary study database by 1 of the study investigators (J.W.S.). In adverse events with multiple HPDs, these concurrent summaries were used by the investigation study panel to designate an initiating (primary) HPD and contributing (secondary) HPDs that followed from the primary HPD or secondarily led to the adverse event.

### Statistical Analysis

This was a prospective observational study to characterize the incidence of human error in surgical adverse events. In this context, a sample size analysis was not performed, as there were no data to indicate baseline rates of human error in our surgical caseload. Case-specific elements collected in our database included the clinical service volume, adverse event attribution, and the temporal context of adverse events (ie, preoperative, intraoperative, or postoperative). Comparison of the incidence of HPDs between hospitals and service lines was performed using χ^2^ analysis. When calculating incidence of HPDs, HPD was treated as a categorical variable. All analyses were conducted using SAS statistical software version 9.4 (SAS Institute). A 2-sided *P* value less than .05 was considered statistically significant.

## Results

There were 5365 surgical operations performed by the general surgery, acute care surgery, surgical oncology, cardiothoracic surgery, vascular surgery, and abdominal transplant services that were subject to review during the 6-month period. Adverse events occurred in 182 patients undergoing surgical operations (3.4%); adverse events also occurred in 6 patients who were undergoing nonoperative treatments (Table 1). Human performance deficiencies were observed among 106 of 188 adverse events (56.4%), occurring at similar rates as a function of hospital and service (Table 2). A total of 192 HPDs were identified in these HPD events (Table 3), occurring at a mean (SD) rate of 1.8 (0.9) HPDs per HPD event. Discrepancies between the HPD classification proposed by the presenting resident and final classifications made by the study investigator panel following conference discussion occurred for 21 primary HPD classifications (11.2%) and 31 subclassifications (16.5%) of the 188 adverse events. In all but 1 adverse event, these differences represented human error classifications additional to the resident’s initial HPD classification assignments. In the exception, the study panel deemed 1 adverse event to not be associated with an HPD (a surgical site infection in which all protocols were followed).

**Table 2.** Human Performance Deficiencies by Hospital Site and Specialty

Variable	Adverse Events Associated With Human Performance Deficiency, No. (%)		
	No (n = 82)	Yes (n = 106)	Total (N = 188)
Hospital^a^			
A	25 (13.3)	35 (18.6)	60 (31.9)
B	35 (18.6)	55 (29.3)	90 (47.9)
C	22 (11.7)	16 (8.5)	38 (20.2)
Service^b^			
Acute care or trauma surgery	35 (18.6)	50 (26.6)	85 (45.2)
Cardiothoracic surgery	8 (4.3)	4 (2.1)	12 (6.4)
Surgical critical care	5 (2.7)	5 (2.7)	10 (5.3)
General, colorectal, or bariatric surgery	3 (1.6)	5 (2.7)	8 (4.3)
Surgical oncology	23 (12.2)	30 (16.0)	53 (28.2)
Transplant surgery	4 (2.1)	6 (3.2)	10 (5.3)
Vascular surgery	4 (2.1)	6 (3.2)	10 (5.3)

^a^ χ^2^ analysis, *P* = .12.

^b^ Fisher exact test, *P* = .77.

**Table 3.** Incidence of HPDs in Adverse Events

Classification	HPD Type	No. (%) (n = 192^a^)
Class I	Planning or problem solving	55 (28.6)
A	Active mistakes	48 (25.0)
1	Guideline or protocol misapplication	3 (1.6)
2	Knowledge deficit	7 (3.6)
3	Cognitive bias	38 (19.8)
i	Diagnostic	13 (6.8)
ii	Treatment	25 (13.0)
B	Latent mistakes	7 (3.6)
Class II	Execution	98 (51.0)
A	Lack of recognition	36 (18.8)
B	Lack of attention	22 (11.5)
C	Memory lapse	3 (1.6)
D	Technical error	37 (19.3)
Class III	Rules violation	6 (3.1)
A	Ignoring routine or cutting corners	2 (1.0)
B	Optimizing or personal gain	1 (0.5)
C	Situational or time pressure	3 (1.6)
Class IV	Communication	24 (12.5)
A	Absent	13 (6.8)
B	Assumed	8 (4.2)
C	Misinterpreted	3 (1.6)
Class V	Teamwork	9 (4.7)
A	Ill-defined roles or lack of leadership	3 (1.6)
B	Lack of group expertise	2 (1.0)
C	Failure to evaluate progress	4 (2.1)

Abbreviation: HPD, human performance deficiency.

^a^ Total incidence of HPDs exceeds total number of 106 adverse events with associated HPDs because of the occurrence of 2 or more HPDs in some adverse events.

Human performance deficiencies were most commonly observed during the intraoperative phase of surgical care (103 adverse events [54.8%]), followed by the postoperative (50 adverse events [26.6%]) and preoperative (15 adverse events [8.0%], including 6 patients [3.2%] undergoing nonoperative treatment) phases of care. Human performance deficiencies were identified in multiple phases of care in 8 adverse events (4.3%).

Execution (class II) error was the most common category of HPD in adverse events (Table 3), classified in 98 of 192 HPDs (51.0%). Planning or problem solving (class I) error was the next most common category, classified in 55 HPDs (29.3%). Of 3 other major HPD classifications, communication (class IV) error, teamwork (class V) error, and rules violation (class III) error were classified in 24 (12.8%), 9 (4.8%), and 6 (3.2%) HPDs, respectively.

Cognitive HPDs in execution (ie, lack of recognition, lack of attention, or memory lapse [class II.A-C]), classified in 61 HPDs (31.8%), and cognitive bias in care planning or problem solving (class I.A.3), classified in 38 HPDs (19.8%), were together the most common HPD subclassification (Table 3). Technical error (class II.D) (37 HPDs [19.3%]) and lack of recognition (class II.A) (36 HPDs [18.8%]) were the 2 most common single HPD subclassifications. In comparison, systems-related errors (ie, guideline or protocol misapplications [class I.A.1], latent mistakes [class I.B], and rules violations [class III]) were classified in only 16 HPDs (8.3%).

### Isolated and Clustered HPDs

Among 106 adverse events in which HPDs were identified, 53 (50.0%) were associated with only 1 HPD (isolated HPDs), while the remaining 53 (50.0%) were associated with multiple HPDs (clustered HPDs) (eTable 1 in the Supplement). There was a mean (SD) of 2.6 (1.1) HPDs per multi-HPD event in the multi-HPD cohort.

**Figure 1.**
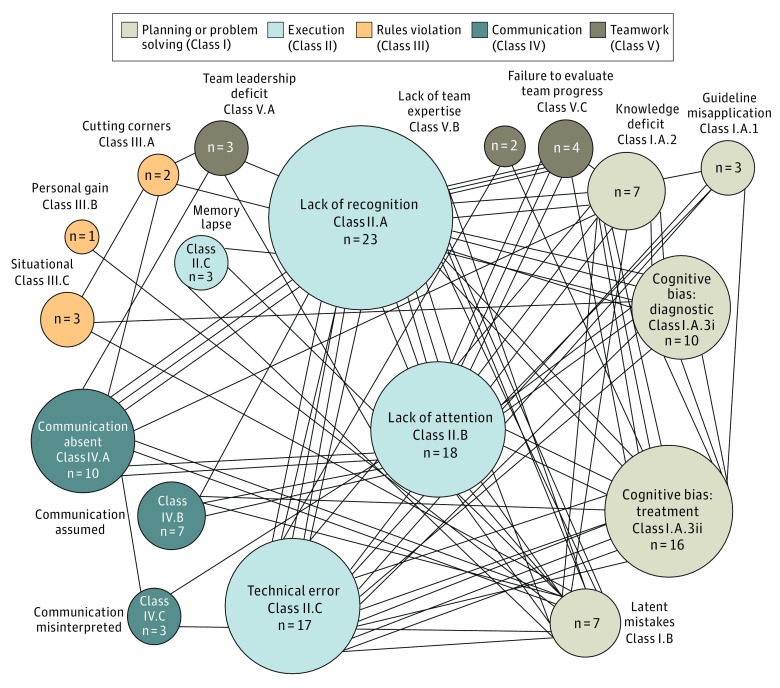
Analysis of Human Performance Deficiency Combinations Among 53 Clustered Human Performance Deficiency Events The size of the circles indicates the frequency of each occurrence of a human performance deficiency. Each line represents 1 association.

Among 53 adverse events with clustered HPDs, the most common association was of technical error (class II.D) with cognitive bias (class I.A.3) (8 HPD events [15.1%]), followed by lack of attention (class II.B) with lack of recognition (class II.A) (5 HPD events [9.4%]), and lack of recognition (class II.A) with technical error (class II.D) (5 HPD events [9.4%]) (Figure 1; eTable 2 in the Supplement). Interestingly, technical error was associated with cognitive error (ie, class I.A.3 [n = 8] or class II.A [n = 5]) in 13 of 17 clustered technical error multi-HPD events (76.5%) and 13 of 37 technical error HPD events overall (35.1%).

### Primary Cause Analysis

**Figure 2.**
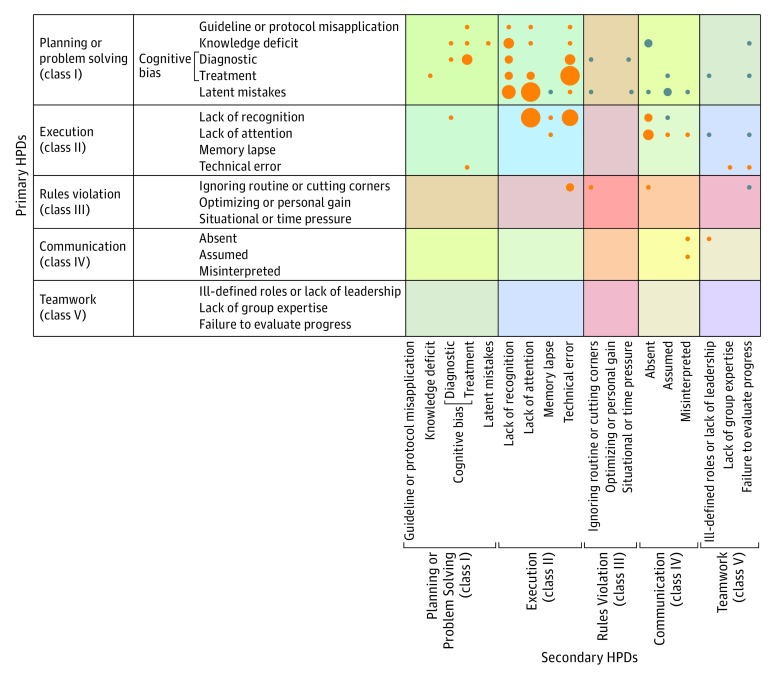
Human Performance Deficiency (HPD) Proximate Cause Analysis Designations were made of a causative HPD (primary HPD) and secondary HPDs in adverse events with clustered HPDs. Orange circles indicate clusters with 1 secondary HPD; blue circles, clusters with 2 or more secondary HPDs. The size of the circles indicates the frequency of occurrence.

A further analysis of the 51 adverse events with clustered HPDs for which a primary HPD was identified (a primary HPD was not identified in 2 multi-HPD events) found that planning or problem solving (class I) was the most common primary HPD category, accounting for secondary HPDs in 29 multi-HPD events (56.9%), and execution (class II) was the primary HPD in 18 multi-HPD events (35.3%) (Figure 2; eTable 3 in the Supplement). Cognitive errors (ie, class I.A.3 or class II.A-C) were the most common primary HPD subclassification, occurring in 31 of 51 multi-HPD events (61.0%) with a primary HPD.

Interestingly, while active planning or problem solving (class I.A) HPDs were found to be secondary HPDs in only 13 of 51 multi-HPD events (25.4%) with a primary HPD (eTable 3 in the Supplement), technical errors (class II.D) were identified as secondary HPDs in 15 of 17 clustered technical error HPD events (88.2%), with cognitive error (class I.A.3 or class II.A-C) identified as the primary HPD of a secondary technical error in all 13 of 17 technical error multi-HPD events (76.5%) with which it was associated. In comparison, other execution errors (class II.A-C) were found to be secondary to other HPDs in 28 of 53 multi-HPD events (50.0%), most commonly with latent mistakes as the primary HPD (class I.B) (10 multi-HPD events [18.9%]), followed by cognitive bias in planning or problem solving (class I.A.3) as the primary HPD (7 multi-HPD events [13.2%]).

## Discussion

This study found that HPDs, or human error, were identifiable in more than half of the complications occurring in major cardiothoracic, vascular, abdominal transplant, surgical oncology, acute care, or general surgical operations performed at 3 large institutions in a major academic medical center. Given that we and others report a current surgical adverse event rate of nearly 5%,^12,13,14,15,17^ our data suggest that more than 400 000 potentially preventable adverse events associated with HPDs occur among the nearly 17 million inpatient and ambulatory operative procedures performed in the United States annually.^20^

Similarities between adverse event rates in our study compared with previous studies suggest that human error remains a significant unresolved cause of adverse events in health care delivery.^4,5,6,7,8,9,13,14,15,16,17,18^ Specifically, compared with our currently reported adverse event rate of 3%, prior studies conducted as early as 2000^7,15,17,21,22,23,24,25,26,27,28,29,30^ reporting a preventable or human error event rate ranging from approximately 3% to 4% support the need for interventions beyond current systems-based strategies if we are to achieve Six Sigma safety levels.

In our current analysis, cognitive error (ie, HPD class I.A.3 and class II.A-C) was the most common specific form of HPD, classified in 99 (52%) of 192 HPDs. More specifically, cognitive error was classified in 29 of 53 isolated HPD events (55%) and 70 of 139 clustered HPD events (50%). It is interesting that lack of recognition was the most prevalent cognitive error and was classified in 19% of the HPD subclassifications, potentially reflecting the paradox that the most common dangers to patient safety are those that are initially unrecognized. This paradox raises important challenges for cognitive training.

Cognitive error was likewise the most common primary cause of adverse events in clustered HPD events, being classified as such in 31 of 53 clustered HPD events (58%). Cognitive error was a potentially preventable primary cause of adverse events in 35% of technical error HPD events. In comparison, technical errors occurring in isolation were classified in only 10% of HPD events, and systems-based (class I.A.1, class I.B, and class III), communications (class IV), or teamwork (class V) HPDs; together represented only 26% of HPD events. These findings suggest the dominant role of cognitive error as a root cause of surgical adverse events, even those that would appear to be technical rather than cognitive in nature.

Considering the relative frequency of HPD types found in our study, the prevalence of cognitive error as the most frequent isolated HPD associated with adverse events may reflect the potency of cognitive error in being able to overwhelm other potential barriers to adverse events, consistent with the Swiss cheese model of the multifactorial causality of human error by Reason.^31^ In comparison, the frequency with which cognitive error was associated with other HPDs (100 of 192 HPDs [52%]) may also reflect the snowballing effect of HPDs, as suggested by Mold and Stein^32^ and Woolf et al.^33^

Two specific examples from our HPD analysis further highlight the potentially unrecognized role that cognitive error can also play in surgical adverse outcomes. In one case, we found that a significant technical error in a surgeon’s performance of an operation was likely precipitated by the initially unappreciated influence of the surgeon’s distraction by an outside telephone call in the operating room. The identification of distraction (ie, class II.B, lack of attention) converts an otherwise challenging technical training goal into an opportunity for behavioral training to reset following intraoperative distractions.

In a second case, a stylus that was inadvertently retained postoperatively was clearly visible but repeatedly unrecognized by radiologists in their reports prior to the patient developing a life-threatening adverse reaction. Standard event analyses would likely ascribe this outcome to clinician inattention, but we were able to further resolve this to confirmation bias (class I.A.3): the clinician dismissed their own concerns because they were not validated in official radiology reports. Like the first example, this HPD subanalysis would allow a cognitive training opportunity to teach clinicians to avoid losing their situational awareness to the convenience of alternative data.

Systems-based approaches (eg, no telephone calls in the operating room, catheter placement checklist) represent standard remedies to these HPD scenarios. However, the effectiveness of these strategies is becoming impaired by a growing checklist burnout syndrome.^12,14,21,22,23,24,34^ The option of cognitive training for health care practitioners as practiced in the aviation industry^19^ also fits with Swiss cheese model by Reason^31^ of multilayered human error safeguards.^10,32,35,36^ As noted by Gruen et al, “protocols alone are insufficient to consistently change behavior.”^15^

### Strengths and Limitations

Our study strengths include contemporaneous engagement of practitioners with firsthand knowledge of index complications, which helped identify first-person details of the practitioner’s thought processes that may have represented cognitive errors or other potentially actionable root-causes. In contrast, prior studies^13,17^ have used retrospective methods, such as interviews and surveys of practitioners months after adverse events have occurred and reports that provide only an overview of potential, relatively broad, nonactionable HPDs without going into deeper analysis. For example, a 2003 study^13^ described outcomes only as system factors, cognitive factors, errors in judgment, inexperience or lack of competence, and fatigue or excessive workload, whereas a 2008 study^17^ that used external investigators without firsthand knowledge of events reported only broad categories of HPDs, including surgical technique, judgment errors, inattention to detail, and incomplete understanding.

Our study is potentially limited in that our HPD adjudication method does not provide absolute certainty as to the primary cause of adverse events, in part because it relies on self-reporting, which in turn must occur in a just culture. Even when HPDs are identified, inherent subjectivity is an additional obstacle to determining a true primary cause of adverse events, but a just culture can provide a productive basis for candidly discussing adverse events and arriving at reasonable assessments of primary causes, as was attempted in our study. In this context, there is an inherent temptation to identify a causal relationship between multiple HPDs that may occur in the same adverse event when no such relationship exists. Likewise, primary cause analyses of adverse events face the challenge of potentially inaccurate attribution, given the post hoc nature of analyzing incompletely interpretable cognitive, team dynamics, and systems-based functions.

It must also be recognized that this study was performed in an academic medical setting and involved a heterogeneous sample of surgical operations that may not be applicable to other institutions with different cultures and case mixes. Nevertheless, consistencies in results among our 3 study sites and across surgical services^15,25,26^ and with national outcomes databases, such as the American College of Surgeons National Surgical Quality Improvement Program,^2,4,6^ suggest that our study does reasonably reflect current real-world institutional deployment of surgical safety safeguards and has potential generalizability.

## Conclusions

Our study contemporaneously applied a newly developed HPD classification system and incorporated practitioner engagement to specifically highlight actionable subtypes of cognitive error as a prevalent source of adverse events in health care delivery. These data suggest that an opportunity exists to develop simulation-based cognitive training of health care practitioners and teams to reinforce systems-based safety constructs, which alone were unable to prevent many adverse events in our study.^19,37,38^ As an example of such training, exercises could involve simulated playbacks of real-life scenarios taken from our situation, background, assessment, and recommendation anthology, similar to training performed in the aviation and aerospace industries. The high intensity of surgical care is particularly sensitive to cognitive error, as reflected in the prevalence of HPDs that we and others have observed to occur during the intraoperative phase of surgery vs the preoperative and postoperative phases in our study and by others.^13,39,40^ Therefore, as a next step, we recommend other academic medical centers and community hospitals similarly study the roles of individual HPDs in adverse outcomes in surgical care delivery to validate the generalizability of these data.

## Notes

Supplement. **eTable 1.** Incidence of Single vs Multiple HPDs **eTable 2.** Associations of HPDs in Multi-HPD Adverse Events **eTable 3.** Primary and Secondary HPD Association Patterns **eFigure 1.** PRISMA Diagram of Literature Analysis **eFigure 2.** Examples for Classification of Human Performance Deficiencies **eFigure 3.** Example of Human Performance Deficiency (HPD) Classifier Tool
