# Supplementary material for: Analysis of Human Performance Deficiencies Associated With Surgical Adverse Events
Source: JAMA Netw Open. 2019 Jul 31;2(7):e198067. doi: 10.1001/jamanetworkopen.2019.8067 (PMC6669897; doi:10.1001/jamanetworkopen.2019.8067)
Supplement: Supplement. — eTable 1. Incidence of Single vs Multiple HPDs eTable 2. Associations of HPDs in Multi-HPD Adverse Events eTable 3. Primary and Secondary HPD Association Patterns eFigure 1. PRISMA Diagram of Literature Analysis eFigure 2. Examples for Classification of Human Performance Deficiencies eFigure 3. Example of Human Performance Deficiency (HPD) Classifier Tool [file jamanetwopen-2-e198067-s001.pdf]

Supplementary Online Content

Suliburk JW, Buck QM, Pirko CJ, et al. Analysis of human performance deficiencies associated with surgical adverse events. *JAMA Netw Open*. 2019;2(7):e198067. doi:10.1001/jamanetworkopen.2019.8067

eTable 1. Incidence of Single vs Multiple HPDs
eTable 2. Associations of HPDs in Multi-HPD Adverse Events
eTable 3. Primary and Secondary HPD Association Patterns
eFigure 1. PRISMA Diagram of Literature Analysis
eFigure 2. Examples for Classification of Human Performance Deficiencies
eFigure 3. Example of Human Performance Deficiency (HPD) Classifier Tool

This supplementary material has been provided by the authors to give readers additional information about their work.

© 2019 Suliburk JW et al. *JAMA Network Open.*

eTable 1. Incidence of Single vs Multiple HPDs

Human Performance Deficiency Classification	Cases with Single HPDs (n = 53)[a]	Cases with Multiple HPDs (n = 53)[a]	Total HPDs
**Class I: Planning/Problem Solving**	**12**	**43**	**55**
A. Active mistakes	12	36	48
1. Guideline/protocol misapplication	0	3	3
2. Knowledge deficit	0	7	7
3. Cognitive bias	12	26	38
i. Diagnostic	3	10	13
ii. Treatment	9	16	25
B. Latent mistakes	0	7	7
**Class II: Execution**	**37**	**61**	**98**
A. Lack of recognition	13	23	36
B. Lack of attention	4	18	22
C. Memory lapse	0	3	3
D. Technical error	20	17	37
**Class III: Rules Violation**	**0**	**6**	**6**
A. Ignoring routine/cutting corners	0	2	2
B. Optimizing/personal gain	0	1	1
C. Situational/time pressure	0	3	3
**Class IV: Communication**	**4**	**20**	**24**
A. Absent	3	10	13
B. Assumed	1	7	8
C. Misinterpreted	0	3	3
**Class V: Teamwork**	**0**	**9**	**9**
A. Ill-defined roles/lack of leadership	0	3	3
B. Lack of team expertise	0	2	2
C. Failure to evaluate progress	0	4	4
**Total Human Performance Deficiencies**	**53**	**139**	**192 (100)****

[a]The number of adverse event cases with single or multiple HPDs

**106 HPD cases noted in column heads is exceeded by 192 HPDs detailed in chart because of the occurrence of two or more HPDs in some adverse event cases (i.e., 1.8 HPDs/adverse event case with HPD).

© 2019 Suliburk JW et al. *JAMA Network Open.*

eTable 2. Associations of HPDs in Multi-HPD Adverse Events

	Associated HPD Major Category	Associated HPD Sub- category (n)
**Class I: Planning/Problem Solving:**		
A. Active mistakes		
1. Guideline/protocol misapplication	Class I, II	IA2, IA3ii (2), IIB, IID, IIB
2. Knowledge deficit	Class I, II, IV, V	IA1, IA3i (2), IA3ii (2), IIA (3), IID, IVA, VC
3. Cognitive bias		
i. Diagnostic	Class I, II, III	IA2 (2), IA3ii (3), IIB, IIA(3), IID(2), IIIA, IIIC
ii. Treatment	Class I, II, IV, V	IA1(2), IA2 (2), IA3i (3), IIA (2), IIB (2), IID (6), IVB, VB, VC
B. Latent mistakes	Class II, III, IV	IIA (4), IIB (4), IIC, IID, IIIB, IIIC, IVA IVB (3), IVC
**Class II: Execution**		
A. Lack of recognition	Class I, II, IV, V	IA2 (3), IA3i (3), IA3ii (2), IB (4), IIB (5), IIC, IID (5), IVA(3), IVB, VA, VC (3)
B. Lack of attention	Class I, II, IV, V	IA1, IA3i, IA3ii (2), IB (4), IIA(5), IIC, IVA, IVB (2) IVC, VA, VC (3)
C. Memory lapse	Class I, II	IB, IIA, IIB
D. Technical error	Class I, II, V	IA1, IA2, IA3i (2), IB, IA3ii (6), IIA (5) VB,
**Class III: Rules Violation**		
A. Ignoring Routine / cutting corners	Class I,III, IV, V	IA3i, IIIC, IVA, VA,
B. Optimizing/personal gain	Class I	IB
C. Necessary/situational	Class I, III	IA3i, IB, IIIA
**Class IV: Communication**		
A. Absent	Class I, II, III IV, V	IA2, IB, IIA(3), IIB (2), IIIA, IVC, VA
B. Assumed	Class I, II, IV	IA3ii, IB (3), IIA, IIB (2), IVC
C. Misinterpreted	Class I, II, IV	IB, IIB, IVA,
**Class V. Teamwork**		
A. Ill-defined roles/lack of leadership	Class II, III, IV	IIA, IIB, IIIA, IVA
B. Lack of group expertise	Class I, II	IA3ii, IID
C. Failure to evaluate progress	Class I, II	IA2, IA3ii, IIA (3), IIB(3),

© 2019 Suliburk JW et al. *JAMA Network Open.*

eTable 3. Primary and Secondary HPD Association Patterns

Primary HPD (n)	Secondary HPD Major Category (n)	Secondary HPD Sub-Category (n)
**Class I: Planning/Problem Solving - 29**<br>A. Active mistakes (23)		
1. Guideline/protocol misapplication (3)	Class II (2)<br>Class I (2)	IID (1), IIB (1)<br>IA3ii (1), IA2 (1)
2. Knowledge deficit (5)	Class II (4)<br>Class I (4)<br>Class IV (1)<br>Class V (1)	IIA (3), IID (1)<br>IA3i (2), IA3ii (1), IB (1)<br>IVA (1)<br>VC (1)
3. Cognitive bias (15)<br>i. Diagnostic (5)	Class II (6)<br>Class III (2)<br>Class I (5)	IIA (2), IIB (1), IID (3)<br>IIIA (1), IIIC (1)<br>IA3ii (3), IA3i (2)
ii. Treatment (10)	Class II (9)<br>Class V (2)<br>Class IV (1)<br>Class I (2)	IID (5), IIA (2), IIB (2)<br>VB (1), VC (1)<br>IVB (1)<br>IA2 (1)
B. Latent mistakes (6)	Class II (10)<br>Class IV (5)<br>Class III (2)	IIA (4), IIB (5), IIC (1)<br>IVA (1), IVB (3), IVC (1)<br>IIIB (1), IIIC (1)
**Class II: Execution - 18**<br>A. Lack of recognition (12)	Class II (11)<br>Class IV (3)<br>Class I (1)	IIB (5), IID (5), IIC (1)<br>IVA (2), IVB (1)<br>IA3i (1)
B. Lack of attention (4)<br>C. Memory lapse (0)	Class IV (5)<br>Class V (2)<br>Class II (1)<br>-	IVA (3), IVB (1), IVC (1)<br>VA (1), VC (1)<br>IIC (1)
D. Technical error (2)	Class V (2)<br>Class I (1)	VB (1), VC (1)<br>IA3ii (1)
**Class III: Rules Violation - 1**<br>A. Ignoring Routine / cutting corners (1)<br>B. Optimizing/personal gain (0)<br>C. Necessary/situational (0)	Class IV (1)<br>Class III (1)<br>Class V (1)<br>-<br>-	IVA (1)<br>IIIC (1)<br>VA (1)
**Class IV: Communication - 3**		
A. Absent (2)	Class V (1)	VA (1)
B. Assumed (1)	Class IV	IVC (1)
C. Misinterpreted (0)	-	
**Class V: Teamwork - 0**<br>A. Ill-defined roles/lack of leadership (0)<br>B. Lack of group expertise (0)<br>C. Failure to evaluate progress (0)	-<br>-<br>-<br>-	

eTable 3 Legend: Primary HPD (orange) column depicts incidence of HPD as the "driving" causative source of clustered (secondary) HPDs in corresponding row. Secondary HPD (turquoise) column depicts frequency at which an HPD was driven by a primary HPD (depicted in orange column of corresponding row)

© 2019 Suliburk JW et al. *JAMA Network Open.*

eFigure 1. PRISMA Diagram of Literature Analysis

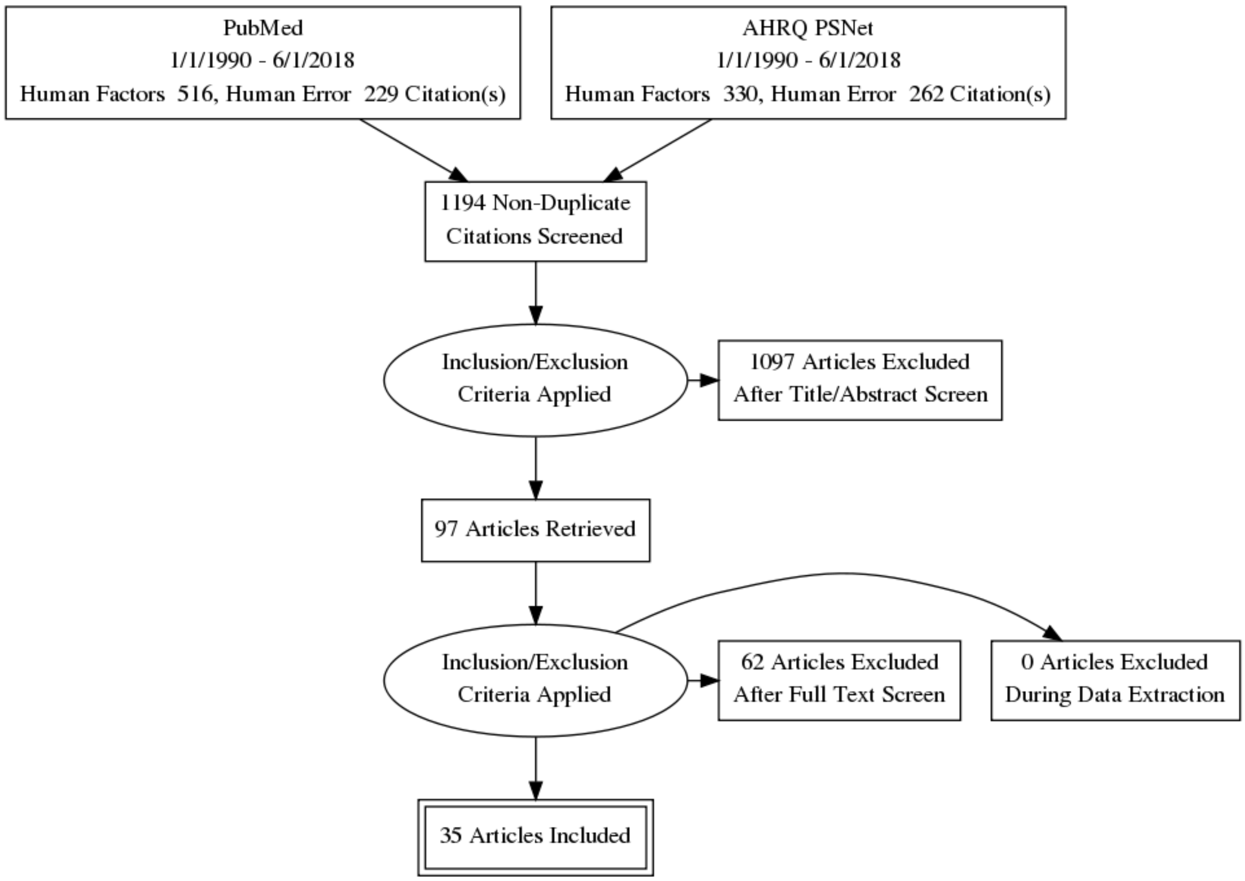

Schematic of systematic review performed using PRISMA guidelines in the synthesis and development of a human performance deficiency (HPD) Classifier Tool.

© 2019 Suliburk JW et al. *JAMA Network Open.*

eFigure 2. Examples for Classification of Human Performance Deficiencies

Human Factors Classification (Examples):

Planning/Problem Solving (confirmation bias, rush to comply, false pattern matching, bias toward action)

A. **Active** Mistake (Proximate cause identifiable with near-immediate consequence)

i. **Rule** based mistakes ("Rule/Protocol" meaning a learned sequence of thoughts or actions meant to address a certain situation)

E.g. - "All free air needs an exploratory laparotomy no matter what" (Bad protocol)

- "Start chest compressions if no pulse in code", but patient just had open heart surgery (Good protocol, wrong situation)

ii. **Knowledge** based mistakes (Novel situation for which previously learned rules/protocols don't work, so have to think of *de novo* solution, but have either incorrect or insufficient base of knowledge or inaccurate logical reasoning)

E.g. - Hypotensive patient with atrial fibrillation given epinephrine as a pressor, but chronotropic effect prompts rapid ventricular response and subsequent decompensation

iii. **Cognitive Bias** in **Diagnosis**

a. Over-attachment to a Diagnosis (E.g. Patient with BRBPR keeps getting exams/treatment for hemorrhoids, delaying colon cancer diagnosis)

b. Availability Bias (E.g. Aortic surgeon assumes new back pain is an acute aortic dissection and doesn't diagnose acute pancreatitis)

c. Diagnostic Momentum (E.g. Postoperative patient with chest pain and dyspnea transferred to ICU with concern for acute MI, so no workup for pulmonary embolism performed)

iv. **Cognitive Bias** in **Treatment Decisions**

a. Commission Bias (E.g. Poor surgical candidate taken for elective ventral hernia repair for symptomatic relief, but malnutrition and poor postoperative healing lead to enterocutaneous fistula)

b. Omission Bias (E.g. Stable cirrhotic patient with incarcerated umbilical hernia isn't taken to OR due to risk of operation, but later perforates and is now too sick for intervention)

B. **Latent** Mistake (**System** factors or **leadership** decisions create a vulnerability which usually lies dormant but was exposed by "perfect storm" of previously unforeseen factors leading to adverse event;

E.g. - Similar color and font labeling on 1:100 and 1:1000 Epi syringes that are stored next to each other

Execution Errors (slips, lapses, fumbles, attention deficit, distraction, situational awareness

A. **Recognition** error (misidentified abnormal patient condition, lab, anatomy, or technical surgical situation)

B. **Attention** error (missed lab, lapsed OR timeout, inattention to anesthesia management)

C. **Memory** error (forgot to write order, forgot patient-specific aberrancy)

D. **Technical** skill/**Insufficient Practice** (Needle travels too deep during fascial closure, causing enterotomy)

Rules Violation (Conscious decision to deviate from safe operating practice, expecting adverse consequences would be avoided)

A. **Routine** ("Cutting corners")

B. **Optimizing**/Personal Gain (Missing afternoon rounds to study or sleep)

C. Necessary/**Situational** (Violation seems to offer only path available or rules seen as inappropriate to situation)

D. **Time** Pressure (Rushing to finish notes, so don't include or edit pertinent details)

Communication

A. **Absent** (Didn't sign out patient's cardiac history)

B. **Assumed** (Intern assumes chief resident meant to give patient double dose of anticoagulant)

C. **Misinterpreted** (Both teams think the other is primary, so no orders get placed)

Teamwork/Group Trap (collective decision/plan is erroneous)

A. **Lack of leadership** in group meeting (roles ill-defined; "I thought you were doing it")

B. No recognition of **lack of subject matter expert** within group (The blind leading the blind)

C. **No pause** to ensure milestones met (Missing the forest for the trees, failing to periodically reevaluate progress of plan)

© 2019 Suliburk JW et al. *JAMA Network Open.*

eFigure 3. Example of Human Performance Deficiency (HPD) Classifier Tool

Human Performance Deficiency Classifier Tool

Procedure ______________________________

Complication ______________________________

○ **Human Factor** Identified ○ **System Factor** Identified ○ **Misdiagnosis** Identified
○ **No Human Factor** Identified ○ **No System Factor** Identified ○ **NO Misdiagnosis** Identified

Error **Timing** (Select **All** That Apply): ○ **Pre-Op** | ○ **Intra-Op** | ○ **Post-Op**

Human Factors Classification (Select **All** That Apply): (See Reverse Page for Explanations/Examples)

I. Planning/Problem Solving (confirmation bias, rush to comply, false pattern matching, bias toward action)

○ A. **Active** Mistake (Proximate cause identifiable with near-immediate consequence)
- ○ i. Rule based mistakes (e.g. effective protocol applied to wrong situation or applied an ineffective protocol)
- ○ ii. Knowledge based mistakes (e.g. misunderstood disease presentation or pathophysiology; misunderstood the mechanism or contraindications of the chosen treatment; fell prey to cognitive biases)
- ○ iii. Cognitive Bias in **Diagnosis**
  - ○ a. Over-attachment to a Diagnosis (Anchoring/Premature Closure/Confirmation Bias)
  - ○ b. Availability Bias (Tendency to repeat diagnoses you've seen recently)
  - ○ c. Diagnostic Momentum (Inheriting other's conclusions without critical analysis)
- ○ iv. Cognitive Bias in **Treatment** Decisions
  - ○ a. Commission Bias (Bias towards beneficence, believing only active intervention can benefit)
  - ○ b. Omission Bias (Bias to avoid maleficence, believing adverse outcomes from natural history are more favorable than those cause by physician action)

○ B. **Latent** Mistake (System factors or leadership decisions create a vulnerability which usually lies dormant but was exposed by "perfect storm" of previously unforeseen factors leading to adverse event)

II. Execution Errors (slips, lapses, fumbles, attention deficit, distraction, situational awareness)

○ A. Lack of Recognition ○ B. Lack of Attention
○ C. Memory Lapse ○ D. Insufficient Practice/Technical Error

III. Rules Violation (Conscious decision to deviate from safe operating practice, expecting adverse consequences would be avoided)

○ A. Routine ("Cutting corners")
○ B. Optimizing/Personal Gain (Rules interfere with personal goals)
○ C. Necessary/Situational (Violation seems to offer only path available or rules seen as inappropriate to situation)
○ D. Time Pressure (Something has to give in order to complete list of tasks)

IV. Communication

○ A. Absent ○ B. Assumed ○ C. Misinterpreted

V. Teamwork/Group Trap (collective decision/plan is erroneous)

○ A. Lack of leadership in group meeting (roles ill-defined; "I thought you were doing it")
○ B. No recognition of lack of subject matter expert within group (The blind leading the blind)
○ C. No pause to reevaluate if milestones are being met or situation has changed (Missing the forest for the trees)

**Narrative Description** (Situation, Background, Assessment, Rx/Results):<br>S –<br>B –<br>A –<br>R –	**Resident** Primary Cause Classification (E.g. [I, A, iii, a])	○ I<br>○ II<br>○ III<br>○ IV<br>○ V	○ A<br>○ B<br>○ C<br>○ D	○ i<br>○ ii<br>○ iii<br>○ iv	○ a<br>○ b<br>○ c
○ differing assessment adjudicated by consensus (if res diff vs attending)<br>○ Failure to Rescue (resulted in mortality/disability)<br>○ Successful Rescue from complication/error (no morality/disability)	**Attending** Primary Cause Classification (E.g. I, A, iii, a)	○ I<br>○ II<br>○ III<br>○ IV<br>○ V	○ A<br>○ B<br>○ C<br>○ D	○ i<br>○ ii<br>○ iii<br>○ iv	○ a<br>○ b<br>○ c

Avatar of human performance deficiency (HPD) Classifier Tool. This document was used by residents and faculty surgeons in the classification and presentation of HPD events. The tool includes examples and subclassifications of HPD events.

© 2019 Suliburk JW et al. *JAMA Network Open.*
